# The role of polyphenol intake and ecological awareness in shaping sustainable nutrition

**DOI:** 10.3389/fnut.2025.1723063

**Published:** 2025-11-13

**Authors:** Hatice Merve Bayram, Arda Ozturkcan

**Affiliations:** Department of Nutrition and Dietetics, Faculty of Health Sciences, Istanbul Gelisim University, Istanbul, Türkiye

**Keywords:** polyphenol intake, Mediterranean diet adherence, sustainable eating, sustainable nutrition, eating behaviors, ecological footprint awareness

## Abstract

**Background:**

This study aimed to examine the relationship between dietary polyphenol intake, adherence to the Mediterranean diet (MD), sustainable and healthy eating behaviors, and ecological footprint awareness among Turkish adults.

**Methods:**

This cross-sectional study was conducted in Istanbul between January–May 2024 with 197 adults. Participants completed a demographic questionnaire, the Mediterranean Diet Adherence Screener (MEDAS), the Sustainable and Healthy Eating Behaviors Scale (SHEB), the Awareness Scale for Reducing Ecological Footprint (ASREF), and a one-day dietary record. Total polyphenol intake was estimated using the Phenol-Explorer database. As the data did not follow a normal distribution, non-parametric tests (Spearman and Kruskal–Wallis) were applied.

**Results:**

A total of 87.8% of participants showed low adherence to the MD. Mean polyphenol intake was 1,616 ± 641 mg/day. Polyphenol intake was moderately positively correlated with MEDAS scores (*r* = 0.456, *p* < 0.001) and weakly correlated with SHEB scores (*r* = 0.147, *p* < 0.05). SHEB and ASREF scores demonstrated a moderate positive correlation (*r* = 0.498, *p* < 0.001). In regression models, ecological footprint awareness emerged as the strongest predictor of sustainable and healthy eating behaviors.

**Conclusion:**

Despite overall low adherence to the MD, higher polyphenol intake was associated with greater compliance. Ecological footprint awareness was the most significant determinant of sustainable dietary practices, highlighting the importance of combining polyphenol-rich foods with strategies that enhance environmental consciousness to promote public health and sustainability.

## Introduction

The extensive use of fossil fuels and other human activities has intensified climate change worldwide, driven by global population growth and industrial expansion (1–3). Sustainable nutrition has become a pivotal idea that connects human well-being with ecological preservation. It is vital to combat climate change and ecological degradation, according to the Food and Agriculture Organization of the United Nations (FAO). This can be achieved by promoting dietary patterns that are both nutritionally adequate and environmentally responsible (4).

Of these dietary habits, the Mediterranean diet (MD) is notable for combining health benefits with ecological sustainability. Consisting of plant-based foods, olive oil, legumes, whole grains, and moderate amounts of fish and poultry (5–7). As well as these main nutrients, the MD is also rich in polyphenols, bioactive compounds derived mainly from fruits, vegetables, olive oil, legumes, nuts, coffee, and red wine, that are responsible for many of its antioxidant and anti-inflammatory effects (8). Previous studies have shown that olive oil, fruits, vegetables, coffee and wine are the foods that contribute most to total dietary polyphenol intake (9, 10). Population-based analyses from Mediterranean cohorts have further demonstrated that individuals with greater adherence to the MD display significantly higher total and class-specific polyphenol intakes (10–13). Moreover, cross-sectional findings indicate that individuals following MD-based diets exhibit greater overall dietary antioxidant capacity, driven primarily by flavonoid and phenolic acid intake (13, 14).

Dietary polyphenols are important contributors to both health and sustainability. These bioactive compounds are abundant in fruits, vegetables, legumes, coffee, and tea. Research has shown that eating polyphenols can help improve health, boost antioxidant levels, and protect against chronic diseases (8, 15, 16). Adherence to MD has been associated with a reduced risk of cardiovascular disease, obesity and type 2 diabetes (5–7). Moreover, recent longitudinal studies have also linked higher total polyphenol intake to reduced incidence of type 2 diabetes and slower biological aging, particularly among older adults adhering to MD-style eating (17, 18).

The MD has been shown to have significant health benefits, in addition to being recognized as a sustainable dietary pattern. It has been indicated by several studies that higher adherence to the MD is associated with lower greenhouse gas emissions, reduced land use, and decreased water footprint compared to Western dietary patterns (1, 19–23). However, studies from Türkiye have reported relatively low adherence to the MD despite growing awareness of the principles of healthy eating (24, 25).

As these foods are mostly plant-based, eating more of them is also in line with sustainable and ecological dietary practices (26). Ecological footprint awareness is the recognition of how individual consumption and lifestyle choices—especially dietary behaviors—affect natural resources and environmental sustainability (27). Recent evidence suggests that individuals with greater ecological awareness are more likely to engage in sustainable dietary practices, such as choosing organic and plant-based foods, which contribute to reducing their ecological footprint (28). However, evidence on how polyphenol intake is related to both MD adherence and sustainable eating behaviors, while simultaneously linked to ecological footprint awareness, remains limited, particularly in the Turkish adult population.

In light of previous evidence highlighting the nutritional and ecological relevance of polyphenol-rich MD, this study aimed to evaluate the relationship between dietary polyphenol intake, adherence to the MD, sustainable and healthy eating behaviors, and ecological footprint awareness among Turkish adults. We hypothesized that individuals with higher polyphenol intake would demonstrate greater adherence to the MD and stronger sustainable and healthy eating behaviors, which in turn would be associated with higher ecological footprint awareness.

## Materials and methods

### Study design and sample

This cross-sectional study was conducted in Istanbul, Türkiye, between January–May 2024, involving adults aged 18–64 years. The sample size was determined using power analysis with an assumed prevalence of 20%, a significance level of 0.05 (*α*), and a power of 80% (1-*β* = 0.80). Based on this calculation, the minimum number of participants required was 100.

A face-to-face questionnaire form including demographic characteristics, Mediterranean Dietary Adherence Scale (MEDAS), Sustainable and Healthy Eating Behaviors Scale (SHEB), Awareness Scale for Reducing Ecological Footprint (ASREF), and one-day food consumption record was applied to the participants. The participants’ height, and body weight values were taken in accordance with their statements. Body mass index (BMI) was calculated as body weight (kg)/height (m)^2^.

Participants were eligible for inclusion if they: were adults aged between 18 and 64 years, resided in Istanbul during the study period, voluntarily agreed to participate and provided written informed consent, were free from conditions requiring regular medication or dietary therapy at the time of the study. Exclusion criteria included: adults with diabetes, cardiovascular disease, kidney or liver disease, thyroid disorders, cancer, those following a special diet, chronic alcohol consumers, menopausal women, and pregnant or lactating women.

### Mediterranean dietary adherence screener

Adherence to the Mediterranean diet was assessed using the MEDAS, originally developed by Schröder et al. (29) and Martínez-González et al. (30), and validated in Turkish by Pehlivanoğlu et al. (24). The screener consists of 14 items, each scored as 0 or 1 depending on compliance with dietary criteria. Total scores were interpreted as follows: <7 = low adherence, 7–8 = moderate adherence, and ≥9 = high adherence (24).

#### Sustainable and healthy eating behaviors scale

Sustainable dietary behaviors were measured using the SHEB scale, developed by Żakowska-Biemans et al. (31), and adapted to Turkish by Köksal et al. (25). The scale consists of 32 items grouped into seven factors (quality labels, seasonal food and food waste avoidance, animal welfare, meat reduction, healthy and balanced diet, local food, and low-fat diet). Items are rated on a seven-point Likert scale ranging from “never” to “always.” Higher scores indicate stronger engagement in sustainable and healthy eating behaviors (25).

#### Awareness scale for reducing ecological footprint

Ecological footprint awareness was assessed using the ASREF, developed by Tekindal et al. (27). The scale comprises 30 items across six dimensions (energy, law/regulation, recycling, transportation, water use, and food consumption). Items are scored on a five-point Likert scale, with higher scores reflecting stronger awareness and tendency toward reducing ecological footprint (27).

### Dietary assessment

Dietary intake was assessed through a one-day food record. Participants were asked to document all foods and beverages consumed, including portion sizes, which were estimated using a validated photographic food atlas (32). For packaged products, brand names and portion weights were recorded, and nutrient contents were cross-verified. Daily energy and nutrient intake were analyzed using the Turkish version of the BeBiS 9 software (Willstätt, Germany).

### Estimation of total polyphenol intake

Total dietary polyphenol intake was estimated using the Phenol Explorer database (version 3.6; accessed December 25, 2024) (33), using data determined by the Folin–Ciocalteu assay, expressed as mg of gallic acid equivalents (GAE) per 100 g of edible portion. When multiple analytical data were available for a given food, mean values reported across studies were used to ensure consistency, as recommended by Phenol-Explorer methodology (34, 35). The Phenol-Explorer database compiles standardized compositional data from over 80 analytical studies covering fruits, vegetables, cereals, legumes, beverages, and other Mediterranean diet components. Although the database has not been updated since 2015, it remains the most comprehensive and standardized reference source for estimating total dietary polyphenol intake across population-based studies.

To allow for comparative analyses, participants were divided into tertiles according to the distribution of total polyphenol intake within the study population: T1 (<25th percentile), T2 (25th–75th percentile), and T3 (>75th percentile). The corresponding cut-off values were <1238.74 mg/day for T1, 1238.74–1784.42 mg/day for T2, and >1784.42 mg/day for T3.

### Statistical analysis

Data were analyzed using SPSS version 24.0. Categorical variables were presented as frequencies and percentages, while continuous variables were reported as mean ± standard deviation (SD). Normality of distribution was tested with the Kolmogorov–Smirnov test. Chi-square tests were used for categorical variables, and Kruskal-Wallis test was used for continuous variables. The Spearman correlation test was used to assess the relationships among age, BMI, total polyphenol intake, and the MEDAS, SHEB, and ASREF scores. Associations between polyphenol intake tertiles and MEDAS, SHEB, and ASREF scores were examined using multivariate logistic regression analysis. A *p*-value ≤0.05 was considered statistically significant.

## Results

Demographic characteristics of the participants are shown in Table 1. The mean age of participants was 25 ± 7 years, and 79.2% were female. Regular physical activity was reported by 25.4% of the participants, while 55.8% reported regular sleep. The mean BMI was 23 ± 4 kg/m^2^. The mean daily polyphenol intake was 1,616 ± 641 mg. Mean scores for MEDAS, SHEB, and ASREF were 6 ± 2, 125 ± 36, and 112 ± 27, respectively.

**Table 1:** Demographic characteristics (*n* = 197).

Parameters	n	%
Age (year) (mean ± SD)	25 ± 7	
Gender [*n* (%)]		
Male	41	20.8
Female	156	79.2
Education status [*n* (%)]		
Below High school	2	1.0
High school and above	195	99.0
Regular physical activity [*n* (%)]		
No	147	74.6
Yes	50	25.4
Regular sleep [*n* (%)]		
No	87	44.2
Yes	110	55.8
Presence of a chronic disease [*n* (%)]		
No	167	84.8
Yes	30	15.2
Height (cm) (mean ± SD)	167 ± 8	
Body weight (kg) (mean ± SD)	65 ± 15	
BMI (kg/m^2^) (mean ± SD)	23 ± 4	
Polyphenol intake (mg/d) (mean ± SD)	1,616 ± 641	
MEDAS scores (mean ± SD)	6 ± 2	
SHEB scale scores (mean ± SD)	125 ± 36	
ASREF scores (mean ± SD)	112 ± 27	

BMI: Body mass index, MEDAS: Mediterranean Dietary Adherence Screener, SHEB: Sustainable and Healthy Eating Behaviors Scale, ASREF: Awareness Scale for Reducing Ecological Footprint.

Table 2 presents the distribution of participants according to daily polyphenol intake tertiles and Mediterranean diet adherence categories. Half of the participants (50.2%) were in the Tertile 2. Based on MEDAS classification, low adherence was the most common (87.8%), while only 0.5% demonstrated high adherence to the Mediterranean diet.

**Table 2:** Mean daily polyphenols intake and Mediterranean diet adherence of the participants (*n* = 197).

Parameters	*n*	%
Polyphenol consumption per day		
Tertile 1 (<1238.74)	49	24.9
Tertile 2 (1238.11–1784.42)	99	50.2
Tertile 3 (>1784.42)	49	24.9
MEDAS classification		
Low MDA (<7 scores)	173	87.8
Moderate MDA (7–9 scores)	23	11.7
High MDA (≥9 scores)	1	0.5

MEDAS: Mediterranean Dietary Adherence Screener, MDA: Mediterranean Diet Adherence.

Table 3 shows the comparison of selected characteristics across tertiles of daily polyphenol intake. MEDAS scores differed significantly among tertiles (*p* < 0.001), with higher intake associated with greater Mediterranean diet adherence. The distribution of MEDAS adherence categories also varied significantly, with a greater proportion of moderate adherence in the highest tertile. No statistically significant differences were observed for gender, age, BMI, SHEB or ASREF scores across tertiles.

**Table 3:** Comparison of selected characteristics according to polyphenol consumption tertiles.

Parameters	Tertile 1 (<1238.74)	Tertile 2 (1238.11–1784.42)	Tertile 3 (>1784.42)	*p*-value
Gender [*n* (%)]				0.520
Male	9 (22.0)	19 (46.3)	13 (31.7)	
Female	40 (25.6)	80 (51.3)	36 (23.1)	
Age	23.0 (21.0–26.5)	23.0 (22.0–28.0)	22.0 (21.0–24.0)	0.090
BMI (kg/m^2^)	23.4 (20.7–25.0)	22.4 (20.6–25.2)	21.97 (20.4–24.7)	0.552
MEDAS score^a,b^	5.0 (4.0–6.0)	5.0 (5.0–6.0)	7.0 (6.0–8.0)	<0.001**
MEDAS classification				<0.001**
Low MDA (<7 scores)	47 (27.2)	94 (54.3)	32 (18.5)	
Moderate MDA (7–9 scores)	2 (8.7)	5 (21.7)	16 (69.6)	
High MDA (≥9 scores)	–	–	1 (100)	
SHEB Scale score	124.0 (87.5–143.5)	130.0 (111.0–154.0)	121.0 (104.0–151.5)	0.121
ASREF score	113.00 (99.0–121.0)	117.00 (102.0–134.0)	113.0 (98.5–124.0)	0.211

**p < 0.001. ^a^Differences between tertile 1 and 3, ^b^ Differences between tertile 2 and 3. *Post-hoc* pairwise comparisons were performed using the Dunn–Bonferroni test following the Kruskal–Wallis analysis. BMI: Body mass index, MEDAS: Mediterranean Dietary Adherence Screener, SHEB: Sustainable and Healthy Eating Behaviors Scale, ASREF: Awareness Scale for Reducing Ecological Footprint. Bold values indicate statistically significant results (**p < 0.001).

Table 4 presents the correlation analysis between age, BMI, total polyphenol intake, and scale scores. Total polyphenol intake showed a moderate positive correlation with MEDAS (*r* = 0.456, *p* < 0.001) and a weak positive correlation with SHEB scores (*r* = 0.147, *p* < 0.05). SHEB scores were moderately correlated with ASREF scores (*r* = 0.498, *p* < 0.001). No significant correlations were found between BMI and either polyphenol intake or MEDAS scores.

**Table 4:** Correlation of the age, BMI, total polyphenol, MEDAS, SHEB, and ASREF scales.

Variables	1	2	3	4	5
1. Age					
2. BMI	0.239**				
3. Total polyphenol	−0.049	0.109			
4. MEDAS	−0.119	−0.033	0.456**		
5. SHEB scale	0.180*	−0.064	0.147*	0.168*	
6. ASREF	0.088	−0.077	0.081	0.127	0.498**

*p < 0.05, **p < 0.001. BMI: Body mass index, MEDAS: Mediterranean Dietary Adherence Screener, SHEB: Sustainable and Healthy Eating Behaviors Scale, ASREF: Awareness Scale for Reducing Ecological Footprint. Bold values indicate statistically significant results (*p < 0.001, **p < 0.001).

Table 5 summarizes the multiple linear regression models predicting SHEB scores. In Model 1, age emerged as a significant predictor (*β* = 0.150, *p* = 0.038). Adding BMI in Model 2 did not improve model fit. In Model 3, total polyphenol intake and age were significant positive predictor (*β* = 0.143, *p* = 0.044, and β = 0.186, *p* = 0.012). Inclusion of MEDAS scores in Model 4 weakened the effect of polyphenol intake, and MEDAS did not show a significant relationship (*β* = 0.160, *p* = 0.052). In the final model, adding ASREF substantially increased the explained variance to 26.5%, with ASREF becoming the strongest predictor (*β* = 0.446, *p* < 0.001).

**Table 5:** Linear regression analysis indicating the predictors of SHEB.

Variables	Unstandardized coefficients		Standardized coefficients						
	**B**	**SE**	**Beta**	**t**	**Sig**	**F**	**Sig**	**R**	**Rsquare effect size**
1 (constant)	89.568	16.616		5.391	<0.001**	2.599	0.077	0.162	0.026
Age	0.822	0.394	0.150	2.087	0.038*				
Gender	8.102	6.416	0.091	1.263	0.208				
2 (constant)	113.516	23.746		4.780	<0.001**	2.402	0.069	0.190	0.036
Age	0.958	0.405	0.175	2.367	0.019*				
Gender	5.451	6.671	0.061	0.817	0.415				
BMI	−0.975	0.692	−0.108	−1.408	0.161				
3 (constant)	99.375	24.566		4.045	<0.001**	2.860	0.025*	0.237	0.056
Age	1.021	0.403	0.186	2.536	0.012*				
Gender	6.441	6.635	0.072	0.971	0.333				
BMI	−1.077	0.688	−0.119	−1.564	0.119				
Total Polyphenol	0.008	0.004	0.143	2.029	0.044*				
4 (constant)	88.657	24.995		3.547	<0.001**	3.087	0.011*	0.273	0.075
Age	1.009	0.400	0.184	2.525	0.012*				
Gender	5.473	6.606	0.061	0.829	0.408				
BMI	−1.014	0.684	−0.112	−1.482	0.140				
Total Polyphenol	0.003	0.005	0.059	0.720	0.472				
MEDAS	3.455	1.766	0.160	1.957	0.052				
5 (Constant)	17.122	24.556		0.697	0.487	11.416	<0.001**	0.515	0.265
Age	0.606	0.362	0.111	1.675	0.096				
Gender	8.679	5.921	0.097	1.466	0.144				
BMI	−0.457	0.617	−0.051	−0.740	0.460				
Total Polyphenol	0.004	0.004	0.068	0.918	0.360				
MEDAS	2.575	1.583	0.119	1.627	0.105				
ASREF	0.599	0.085	0.446	7.012	<0.001**				

*p < 0.05, **p < 0.001. R^2^_adj values ranged from 0.02 to 0.26 across Models 1–5. Assumption checks confirmed no multicollinearity (Variance Inflation Factor [VIF] range = 1.03–1.48) and acceptable model fit. All models met linearity, independence, and homoscedasticity assumptions. BMI: Body mass index, MEDAS: Mediterranean Dietary Adherence Screener, SHEB: Sustainable and Healthy Eating Behaviors Scale, ASREF: Awareness Scale for Reducing Ecological Footprint.

## Discussion

This study demonstrated that, although most participants showed low adherence to the MD, a higher intake of dietary polyphenols was significantly associated with greater adherence to this dietary pattern. Furthermore, polyphenol intake was modestly positively related to sustainable and healthy eating behaviors, while ecological footprint awareness was found to be the strongest predictor of such behaviors in multivariate models. These findings suggest that, while dietary polyphenols may act as a nutritional bridge between individual health and sustainability, ecological awareness plays a more central role in shaping sustainable dietary practices among Turkish adults.

A sustainable diet should be nutritionally adequate and safe, as well as being respectful of biodiversity and ecosystems. It should also be culturally acceptable and accessible (36). It is known that more natural resources are used and a greater negative impact on the environment is had by animal-based foods than plant-based foods. For this reason, the MD is considered the most sustainable dietary model. Its diversity, freshness, seasonality, culture and adherence to tradition have been shown to have positive effects on health (37, 38). Evidence further indicates that higher levels of knowledge regarding sustainable nutrition are associated with stronger adherence to the MD (39). Similarly, adults who perceive their diet as more sustainable are more likely to demonstrate high compliance with the Mediterranean dietary pattern (40). Recent findings also show that individuals with greater adherence to the MD achieve higher SHEB scores. For example, a study found that most individuals adhered to the MD, and those with the highest adherence had higher SHEB scores (1). Consistent with these findings, individuals with elevated SHEB scores have also been reported to show stronger adherence to the MD (41). Moreover, the present study revealed that 87.8% of participants exhibited low adherence to the MD. However, individuals with higher adherence demonstrated increased SHEB scores, indicating a positive relationship between MD compliance and sustainable eating practices, which suggests that adopting the MD pattern may serve as a practical pathway to promote more sustainable nutrition behaviors.

Moreover, polyphenols, a complex group of phytochemicals found abundantly in the MD, contribute significantly to its health-promoting effects (8). Previous research consistently supports the strong link between adherence to the MD and higher total polyphenol intake, particularly in Mediterranean cohorts (11, 12). Early compositional analyses using the Phenol-Explorer database further identified olive oil, fruits, vegetables, coffee, and wine as major contributors to total dietary polyphenols (9, 11), demonstrating that polyphenols represent a biochemical hallmark of the MD. Our results showed that greater dietary polyphenol intake was significantly associated with higher MD adherence, supporting the role of polyphenols as a nutritional bridge between health and sustainability. Although polyphenol intake showed only a moderate association with SHEB, ecological footprint awareness emerged as the strongest determinant of sustainable dietary behaviors in multivariate models.

Increasing vegetable intake and reducing the consumption of animal-based products are dietary shifts that have been associated with reductions in ecological footprint (42). Research indicates that consumers who are aware of their ecological footprint are more likely to adopt sustainable eating behaviors, including higher adherence to the MD, which is celebrated for its lower environmental impact compared to other dietary patterns. For instance, Kabasakal Cetin et al. found that increased awareness of ecological footprint reduction behaviors was positively associated with adherence to the MD, as well as climate-friendly food choices (43) Moreover, Yardımcı and Demirer found that awareness of ecological sustainability can simultaneously elevate the quality of dietary choices, promoting a holistic approach to resource consumption (44). Our previous study showed that there was a moderate positive correlation between the total ecological footprint awareness score and the total sustainable and healthy eating behaviors score (42). In this study we found that, SHEB scores were moderately correlated with ASREF scores, whereas there was no significant correlations between MEDAS and ASREF scores. This may imply that ecological footprint awareness influences broader sustainable dietary practices rather than directly translating into adherence to a specific dietary pattern such as the MD. The strong predictive role of ecological awareness in sustainable dietary behavior may stem from the interplay of knowledge, attitudes, and social influences. Individuals with greater ecological consciousness, often those adhering more closely to sustainable dietary patterns like the MD, tend to prefer environmentally labeled, plant-based, and locally sourced foods (39, 43). Heightened awareness also promotes mindful consumption, encouraging reductions in meat intake and food waste, while creating a self-reinforcing cycle in which awareness and behavior strengthen each other (45). Educational initiatives that integrate sustainability concepts into curricula have been shown to improve understanding of the environmental impact of dietary habits, particularly among university students (46). Moreover, social and cultural norms emphasizing environmental values foster collective motivation toward eco-friendly eating patterns (47). These mechanisms illustrate that ecological awareness is a multidimensional driver, rooted in environmental knowledge, mindful attitudes, and shared values, that reinforces commitment to sustainable dietary practices.

This study has several strengths. It is among the few to simultaneously evaluate dietary polyphenol intake, Mediterranean diet adherence, sustainable and healthy eating behaviors, and ecological footprint awareness in a Turkish adult population. The use of validated measurement tools (MEDAS, SHEB, and ASREF) and a standardized food composition database (Phenol-Explorer) enhances the reliability of the findings.

However, several limitations should be acknowledged. The cross-sectional design precludes causal inferences. Moreover, dietary intake was assessed using a single-day food record, which may not fully capture interday variations in food consumption and polyphenol intake. Self-reported anthropometric and dietary data may also be subject to recall or reporting bias. Although the Phenol-Explorer database (version 3.6) remains the most comprehensive and standardized source for estimating dietary polyphenol intake, it has not been updated since 2015, which may limit the inclusion of newer food composition data. Despite these limitations, the study provides important insights into the interplay between nutritional quality, sustainability, and ecological awareness.

## Conclusion

This study showed that despite the predominance of low adherence to the Mediterranean diet, higher polyphenol intake was associated with improved compliance, underscoring its potential contribution to healthier and more sustainable dietary patterns. Furthermore, while a lower association was shown with sustainable eating behaviors by polyphenol intake, the strongest determinant was identified as ecological footprint awareness, emphasising the importance of environmental consciousness in the shaping of sustainable nutrition practices. These findings suggest that promoting polyphenol-rich, plant-based foods alongside targeted educational strategies aimed at raising ecological awareness could be an effective way of improving both public health and environmental sustainability. In practice, integrating polyphenol-rich food choices and ecological awareness modules into nutrition education programs may enhance sustainable dietary behaviors among adults. Further longitudinal and intervention studies are needed to confirm these associations and explore effective ways of incorporating ecological awareness into nutrition education and policy.

## Data Availability

The original contributions presented in the study are included in the article/supplementary material, further inquiries can be directed to the corresponding author/s.

## References

[1] Kürklü NS Karaçİl Ermumcu MŞ Suna G Özyıldırım C Tel Adigüzel K Aydın M . Adherence to the Mediterranean diet is associated with sustainable nutrition and environmental footprints on higher educated individuals. Int J Environ Res Public Health. (2024) 34:3478–88. doi: 10.1080/09603123.2024.2308732, PMID: 38254327

[2] Bayram HM Ozturkcan SA. Greenhouse gas emissions in the food system: current and alternative dietary scenarios. Mediterr J Nutr Metab. (2022) 15:463–77. doi: 10.3233/MNM-220006

[3] Bayram HM Ozturkcan A. The greenhouse gas emissions from food consumption in Turkey: a regional analysis with developmental parameters. Sustain Food Technol. (2023) 1:92–9. doi: 10.1039/D2FB00027J

[4] Food and Agriculture Organization of the United Nations (FAO). (2019). Sustainable healthy diets – guiding principles. Published 2019. Available online at: http://www.fao.org/3/ca6640en/ca6640en.pdf (Accessed August 20, 2025)

[5] Godos J Guglielmetti M Ferraris C Frias-Toral E Domínguez Azpíroz I Lipari V . Mediterranean diet and quality of life in adults: A systematic review. Nutrients. (2025) 17:577. doi: 10.3390/nu17030577, PMID: 39940436 PMC11819740

[6] Dobroslavska P Silva ML Vicente F Pereira P. Mediterranean dietary pattern for healthy and active aging: A narrative review of an integrative and sustainable approach. Nutrients. (2024) 16:1725. doi: 10.3390/nu16111725, PMID: 38892658 PMC11174674

[7] Xiao Y Xiao X Zhang X Yi D Li T Hao Q . Mediterranean diet in the targeted prevention and personalized treatment of chronic diseases: evidence, potential mechanisms, and prospects. EPMA J. (2024) 15:207–20. doi: 10.1007/s13167-024-00360-w, PMID: 38841625 PMC11147989

[8] Bayram HM Majoo FM Ozturkcan A. Polyphenols in the prevention and treatment of non-alcoholic fatty liver disease: an update of preclinical and clinical studies. Clinical Nutrition ESPEN. (2021) 44:1–14. doi: 10.1016/j.clnesp.2021.06.026, PMID: 34330452

[9] Saura-Calixto F Serrano J Goñi I. Intake and bioaccessibility of total polyphenols in a whole diet. Food Chem. (2017) 101:492–501. doi: 10.1016/j.foodchem.2006.02.006

[10] Tresserra-Rimbau A Medina-Remón A Pérez-Jiménez J Martínez-González MA Covas MI Corella D . Dietary intake and major food sources of polyphenols in a Spanish population at high cardiovascular risk: the PREDIMED study. Nutr Metab Cardiovasc Dis. (2013) 23:953–9. doi: 10.1016/j.numecd.2012.10.008, PMID: 23332727

[11] Godos J Rapisarda G Marventano S Galvano F Mistretta A Grosso G. Association between polyphenol intake and adherence to the Mediterranean diet in Sicily, southern Italy. NFS Journal. (2017) 8:1–7. doi: 10.1016/j.nfs.2017.06.001

[12] Tresserra-Rimbau A Guasch-Ferré M Salas-Salvadó J Toledo E Corella D Castañer O . Intake of total polyphenols and some classes of polyphenols is inversely associated with diabetes in elderly people at high cardiovascular disease risk. J Nutr. (2016) 146:767–77. doi: 10.3945/jn.115.223610, PMID: 26962181

[13] Hernandez-Ruiz A Garcia-Villanova B Guerra-Hernandez E. Comparison of the dietary antioxidant profiles of 21 a priori defined Mediterranean diet indexes. J Acad Nutr Diet. (2018) 118:2254–68. doi: 10.1016/j.jand.2018.01.006 29580874

[14] Louro T Castelo PM Simões C Capela e Silva F Luís H Moreira P . Adherence to mediterranean diet and aromatic plants intake are related with gustatory function: A case-study from a Portuguese region. Appetite. (2024) 201:107581. doi: 10.1016/j.appet.2024.107581, PMID: 38945368

[15] Bayram HM Eren F Gunes FE. The relationship between polyphenols and mi RNAs: a novel therapeutic strategy for metabolic associated fatty liver disease. Hepatology Forum. (2021) 2:128–36. doi: 10.14744/hf.2021.2021.0037 35784906 PMC9138948

[16] Nemzer BV Al-Taher F Kalita D Yashin AY Yashin YI. Health-improving effects of polyphenols on the human intestinal microbiota: A review. Int J Mol Sci. (2025) 26:1335. doi: 10.3390/ijms26031335, PMID: 39941107 PMC11818678

[17] Esposito S Gialluisi A Costanzo S di Castelnuovo A Ruggiero E de Curtis A . Mediterranean diet and other dietary patterns in association with biological aging in the Moli-sani study cohort. Clin Nutr. (2022) 41:1025–33. doi: 10.1016/j.clnu.2022.02.023 35390726

[18] Kaplan A Zelicha H Meir AY Yaskolka Meir A Rinott E Tsaban G . The effect of a high-polyphenol Mediterranean diet (green-MED) combined with physical activity on age-related brain atrophy: the dietary intervention randomized controlled trial polyphenols unprocessed study (DIRECT PLUS). Am J Clin Nutr. (2022) 115:1270–81. doi: 10.1093/ajcn/nqac001, PMID: 35021194 PMC9071484

[19] Dernini S Berry EM. Mediterranean diet: from a healthy diet to a sustainable dietary pattern. Front Nutr. (2015) 2:15. doi: 10.3389/fnut.2015.00015, PMID: 26284249 PMC4518218

[20] Sáez-Almendros S Obrador B Bach-Faig A Serra-Majem L. Environmental footprints of Mediterranean versus Western dietary patterns: beyond the health benefits of the Mediterranean diet. Environ Health. (2013) 12:118. doi: 10.1186/1476-069X-12-118, PMID: 24378069 PMC3895675

[21] Álvarez-Álvarez L Vitelli-Storelli F Rubín-García M García S Bouzas C Ruíz-Canela M . Impact of mediterranean diet promotion on environmental sustainability: a longitudinal analysis. Public Health. (2024) 230:12–20. doi: 10.1016/j.puhe.2024.02.010, PMID: 38479163

[22] Lorca-Camara V Bosque-Prous M Bes-Rastrollo M O'Callaghan-Gordo C Bach-Faig A. Environmental and health sustainability of the Mediterranean diet: a systematic review. Adv Nutr. (2024) 15:100322. doi: 10.1016/j.advnut.2024.100322, PMID: 39426729 PMC11605453

[23] Metin ZE Çelik ÖM Koç N. Relationship between adherence to the Mediterranean diet, sustainable and healthy eating behaviors, and climate change awareness: a cross-sectional study from Turkey. Nutrition. (2024) 118:112266. doi: 10.1016/j.nut.2023.112266, PMID: 37988926

[24] Pehlivanoğlu EFÖ Balcıoğlu H Ünlüoğlu İ. Akdeniz diyeti bağlılık ölçeği’nin Türkçe’ye uyarlanması, geçerlilik ve güvenilirliği. Osmangazi Tıp Dergisi. (2020) 42:160–4. doi: 10.20515/otd.504188

[25] Köksal E Bilici S Dazıroğlu MEÇ Erdoğan Gövez N. Validity and reliability of the Turkish version of the sustainable and healthy eating behaviors scale. Br J Nutr. (2022) 129:1398–404. doi: 10.1017/S0007114522002525 35938236

[26] Bayram HM Aydın AG Okur H Karalı AE Öztürkcan A. The relationship between dietary polyphenol intake and adherence to the Mediterranean diet, mental health, and sleep quality among Turkish adults: A cross-sectional study. Food Health. (2024) 10:262–72. doi: 10.3153/FH24025

[27] Tekindal MA Zabzun G Özel Z Demirsöz M Tekindal M. Awareness scale for reducing ecological footprint: a validity and reliability study. Avrupa Bilim ve Teknoloji Dergisi. (2021) 27:439–45. doi: 10.31590/ejosat.944221

[28] Nichifor B Zaiț L Timiras L. Drivers, barriers, and innovations in sustainable food consumption: a systematic literature review. Sustainability. (2025) 17:2233. doi: 10.3390/su17052233

[29] Schröder H Fito M Estruch R Martínez-González MA Corella D Salas-Salvadó D . A short screener is valid for assessing Mediterranean diet adherence among older Spanish men and women. J Nutr. (2011) 141:1140–5. doi: 10.3945/jn.110.135566, PMID: 21508208

[30] Martínez-González MÁ Corella D Salas-Salvadó J Ros E Covas MI Fiol CM . Cohort profile: design and methods of the PREDIMED study. Int J Epidemiol. (2012) 41:377–85. doi: 10.1093/ije/dyq250 21172932

[31] Żakowska-Biemans S Pieniak Z Kostyra E Gutkowska K. Searching for a measure integrating sustainable and healthy eating behaviors. Nutrients. (2019) 11:95. doi: 10.3390/nu11010095, PMID: 30621258 PMC6357150

[32] Rakıcıoğlu N Tek Acar N Ayaz A Pekcan G. (ed). Yemek ve Besin Fotoğraf Kataloğu-Ölçü ve Miktarlar, 2. Baskı. Ankara: Ata Ofset Matbaacılık (2025).

[33] Phenol-Explorer. (2025). Phenol-explorer: Database on polyphenol content in foods. Paris, France: INRAE. Available online at: https://phenol-explorer.eu (Accessed June 10, 2025).

[34] Rothwell JA Perez-Jimenez J Neveu V Medina-Remón A M’hiri B García-Lobato P . Phenol-explorer 3.0: a major update of the phenol-explorer database to incorporate data on the effects of food processing on polyphenol content. Database. (2013) 2013:bat070. doi: 10.1093/database/bat070, PMID: 24103452 PMC3792339

[35] Neveu V Perez-Jiménez J Vos F Crespy V du Chaffaut L Mennen L . Phenol-explorer: an online comprehensive database on polyphenol contents in foods. Database. (2010) 2010:bap024. doi: 10.1093/database/bap024, PMID: 20428313 PMC2860900

[36] Martinelli SS Cavalli SB. Healthy and sustainable diet: a narrative review of the challenges and perspectives. Ciencia Saúde Coletiva. (2019) 24:4251–62. doi: 10.1590/1413-812320182411.30572017 31664397

[37] Alsaffar AA. Sustainable diets: the interaction between food industry, nutrition, health and the environment. Food Sci Technol Int. (2016) 22:102–11. doi: 10.1177/1082013215572029, PMID: 25680370

[38] World Health Organization. (2019). Sustainable healthy diets – Guiding principles. Geneva, Switzerland: World Health Organization. Available online at: https://www.who.int/publications/i/item/9789241516648 (Accessed September 20, 2025).

[39] Yassıbaş E Bölükbaşı H. Evaluation of adherence to the Mediterranean diet with sustainable nutrition knowledge and environmentally responsible food choices. Front Nutr. (2023) 10:1158155. doi: 10.3389/fnut.2023.1158155, PMID: 37125040 PMC10130392

[40] Biasini B Rosi A Menozzi D Scazzina F. Adherence to the Mediterranean diet in association with self-perception of diet sustainability, anthropometric and sociodemographic factors: a cross-sectional study in Italian adults. Nutrients. (2021) 13:3282. doi: 10.3390/nu13093282, PMID: 34579159 PMC8468784

[41] Kocaadam-Bozkurt B Bozkurt O. Relationship between adherence to the Mediterranean diet, sustainable and healthy eating behaviors, and awareness of reducing the ecological footprint. Int J Environ Health Res. (2023) 33:430–40. doi: 10.1080/09603123.2023.2172384, PMID: 36726049

[42] Bayram HM Ozturkcan A. Eco-anxiety, organic food consumption, and ecological awareness as determinants of sustainable eating behaviors in adults. Mediterr J Nutr Metab. (2025). [In press]. doi: 10.1177/1973798X251377713

[43] Kabasakal Cetin A Şen G Aksaray B. Examining the association of climate change worry and awareness of ecological footprint reduction behaviours with Mediterranean diet adherence and climate-friendly food choices. Br Food J. (2025) 127:168–81. doi: 10.1108/BFJ-06-2024-0577

[44] Yardimci H Demirer B. Is high adaptation to the Mediterranean diet effective in increasing ecological footprint awareness? A cross-sectional study from Turkey. J Sci Food Agric. (2022) 102:3724–9. doi: 10.1002/jsfa.11720, PMID: 34907547

[45] Yücel S Metin ZE. The interplay between mindful eating, climate change awareness, and psychological well-being: A cross-sectional analysis. Food Sci Nutr. (2025) 13:e70716. doi: 10.1002/fsn3.70716, PMID: 40831943 PMC12358938

[46] Lee Y Kim T Jung H. Effects of university students’ perceived food literacy on ecological eating behavior towards sustainability. Sustainability. (2022) 14:5242. doi: 10.3390/su14095242

[47] Straupaite S. The expression of ecological awareness in the consumption behavior of older adolescents E3S Web Conf EDP Sciences (2023) 436:06002 doi: 10.1051/e3sconf/202343606002

